# Effects of Cranberry Extract (*Vaccinium macrocarpon*) Supplementation on Lipid Peroxidation and Inflammation in Patients with Chronic Kidney Disease (Stages 3-4): A Randomized Controlled Trial

**DOI:** 10.1155/2024/9590066

**Published:** 2024-05-08

**Authors:** Laís de Souza Gouveia Moreira, Karla Thaís Resende Teixeira, Ludmila F. M. F. Cardozo, Livia Alvarenga, Bruna Regis, Jessyca Sousa de Brito, Viviane de Oliveira Leal, Natalia Alvarenga Borges, Isabela de Souza da Costa Brum, José Carlos Carraro-Eduardo, Giovanna B. Borini, Andresa A. Berretta, Marcelo Ribeiro-Alves, Denise Mafra

**Affiliations:** ^1^Graduate Program in Medical Sciences, Fluminense Federal University (UFF), Niterói, Rio de Janeiro, Brazil; ^2^Graduate Program in Nutrition Sciences, Fluminense Federal University (UFF), Niterói, Rio de Janeiro, Brazil; ^3^Graduate Program in Cardiovascular Sciences, Fluminense Federal University (UFF), Niterói, Rio de Janeiro, Brazil; ^4^Graduate Program in Biological Sciences -Physiology, Federal University of Rio de Janeiro (UFRJ), Rio de Janeiro, Brazil; ^5^Nutrition Division, Pedro Ernesto University Hospital, Rio de Janeiro State University (UERJ), Rio de Janeiro, Brazil; ^6^Institute of Nutrition, Rio de Janeiro State University (UERJ), Rio de Janeiro, Brazil; ^7^Faculty of Medicine, Fluminense Federal University (UFF), Niterói, Rio de Janeiro, Brazil; ^8^Research, Development & Innovation Department, Apis Flora Industrial e Comercial Ltda., Ribeirão Preto, São Paulo, Brazil; ^9^HIV/AIDS Clinical Research Center, National Institute of Infectology (INI/Fiocruz), Rio de Janeiro, Brazil

## Abstract

**Background:**

Growing evidence suggests that bioactive compounds in berry fruits may mitigate inflammation in patients with chronic kidney disease (CKD).

**Objectives:**

To evaluate cranberry (*Vaccinium macrocarpon*) supplementation effects on modulation of transcription factors involved in inflammation and oxidative stress in nondialysis (stages 3 and 4) patients with CKD. *Design/Participants*. A randomized, double-blind, placebo-controlled study was performed with 30 patients to receive capsules containing cranberry extract (1000 mg/day) or placebo (1000 mg/day of corn starch) for two months. *Measurements*. The mRNA expression of nuclear factor-erythroid 2-related factor-2 (Nrf2) and nuclear factor-kB (NF-kB) was evaluated in peripheral blood mononuclear cells (PBMCs) by quantitative real-time polymerase chain reaction. Thiobarbituric acid reactive substances (TBARS) were measured in the plasma to assess oxidative stress. Interleukin-6 (IL-6) plasma levels were assessed by enzyme-linked immunosorbent assay and C-reactive protein (CRP) by immunoturbidimetric method.

**Results:**

Twenty-five patients completed the study: 12 in the cranberry group (56.7 ± 7.5 years and body mass index (BMI) of 29.6 ± 5.5 kg/m^2^) and 13 in the placebo group (58.8 ± 5.1 years and BMI 29.8 ± 5.4 kg/m^2^). There were no differences in NF-kB or Nrf2 mRNA expressions (*p* = 0.99 and *p* = 0.89) or TBARS, CRP, and IL-6 plasma levels after cranberry supplementation.

**Conclusions:**

The cranberry extract administration (1000 mg/day) did not affect Nrf2 and NF-kB mRNA expression, oxidative stress, or inflammatory markers levels in nondialysis CKD patients. This trial is registered with NCT04377919.

## 1. Introduction

Chronic diseases, also known as noncommunicable diseases, are the leading causes of death and disease burden worldwide and are responsible for 85% of premature deaths in low- and middle-income countries [1]. Among them, chronic kidney disease (CKD) is a health problem with a high mortality rate worldwide [2]. According to the Global Burden of Disease (2017), the global prevalence of CKD is 9.1% (697.5 million cases) [3]. CKD also caused 1.2 million deaths and is the 12th leading cause of mortality worldwide. CKD is projected to be the 5th common cause of loss of life [3, 4].

Patients with CKD have a high cardiovascular risk, which manifests as coronary artery disease, arrhythmia, and heart failure, making cardiovascular diseases the primary cause of death. Traditional risk factors (high blood pressure, obesity, diabetes mellitus, aging, and smoking) associated with nontraditional risk factors, such as mitochondrial and endothelial dysfunction, oxidative stress, and inflammation, contribute to cardiovascular and kidney complications and mortality [5].

Patients with advanced-stage CKD produce a large amount of reactive oxygen species (ROS), which leads to oxidative stress and is associated with several complications. ROS alter protein and DNA integrity, promote lipid peroxidation, affect cellular membrane fluidity, inactivate receptors and enzymes, induce apoptosis, increase tissue permeability, and promote tissue damage [6, 7].

ROS production contributes to the activation of the transcription factor nuclear factor-kappa B (NF-*κ*B) [8], which plays a role in the inflammatory response by stimulating the overproduction of proinflammatory cytokines that also stimulate ROS production [9]. The chronic proinflammatory state also contributes to vascular and myocardial remodeling processes, which promote atherosclerotic lesions, myocardial fibrosis, and vascular calcification, thereby contributing to cardiovascular disease progression [10].

Furthermore, these patients had downregulated levels of transcription factor erythroid nuclear-related factor-2 (Nrf2), which regulates the genes implicated in detoxification and antioxidant capacity [11]. These mechanisms contribute to renal damage and CKD progression, as they are related to the worsening of glomerular damage and renal ischemia, in addition to promoting complications such as inflammation, anemia, endothelial dysfunction, hypertension, and atherosclerosis [12]. Therefore, management of these changes is fundamental for the prevention, treatment, and reduction of CKD progression.

The treatment of nondialysis patients aims to delay the decline in kidney function, manage symptoms, and improve quality of life. Thus, pharmacological interventions and lifestyle modifications are necessary to control this disease, including nutritional designs such as a low-protein diet, reduction of sodium, phosphate, and potassium intake, and adoption of a plant-based diet [13–15].

Bioactive compounds found in plant foods have several properties, such as anti-inflammatory, antioxidant, antiproliferative, and antihypertensive effects, as well as modulation of cardiovascular risk markers [16–19]. Therefore, several researchers have used herbs and natural substances as complementary therapies for chronic diseases and CKD because of their antioxidant, antihypertensive, and anti-inflammatory effects, lipid profile improvement [17], and modulation of transcription factors involved in inflammation and oxidative stress, which can slow CKD progression [20–23]. In this context, the use of bioactive compounds from food [24] (resveratrol, curcumin, sulforaphane, and catechins, among others) has been discussed in the concept of food as medicine for patients with chronic diseases and CKD [25–27].

Cranberry (*Vaccinium* macrocarpon) is extensively studied owing to its chemical composition. It offers a complex variety of bioactive components, including high concentrations of free and bound phenolic compounds, several flavonols, procyanidins, and anthocyanins, and high concentrations of A-type proanthocyanidins (PACs) [28].

Polyphenols in cranberries exert antioxidant effects; however, their exact mechanisms of action are unknown. It can be speculated that polyphenols not only neutralize the production of ROS but also interfere with cell signaling pathways, such as derepressing Nrf2 from Keap1, promoting Nrf2 activation, and promoting NF-*κ*B inhibition as well [29, 30]. Recent reviews have provided mechanistic support for the effects of cranberry on CKD through the modulation of inflammation, oxidative stress, prevention of urinary tract infections (UTIs), and gut microbiota modulation [31, 32]. These studies evaluated different forms of cranberry supplements, such as cranberry powder and juice, under diverse conditions, including diabetes, endothelial dysfunction, cardiometabolic alterations, dyslipidemia, and common CKD complications [32]. However, few studies have evaluated the effects of cranberries on inflammation-related transcription factors. A study using cranberry polyphenolic fractions in intestinal Caco-2/15 cells reported the inactivation of NF-*κ*B and upregulation of Nrf2 [33]. Also, rats supplemented with polyphenol-rich cranberry extract showed lower expression of NF-*ĸ*B [30].

The potential impact of cranberry supplementation on inflammation and oxidative stress in patients with CKD is yet to be investigated in prospective longitudinal studies. In this study, we hypothesized that cranberry supplementation could modulate the expression of transcription factors involved in inflammation, such as Nrf2 and NF-kB, and decrease inflammatory and oxidative stress markers in nondialysis patients with CKD. This study aimed to evaluate the effects of cranberry extract supplementation on the expression of transcription factors involved in inflammation and oxidative stress in nondialysis patients with CKD.

## 2. Materials and Methods

### 2.1. Participants

Here, we present a secondary analysis of previously published studies [34]. Participants were randomized, and the study was performed in pair blocks according to the following equivalent criteria: sex, age, estimated glomerular filtration rate (eGFR), and body mass index (BMI). They were randomized into two study arms: “cranberry group” and “placebo group,” to receive each supplementation. Nondialysis patients with CKD (stages 3 and 4) aged between 20 and 65 years were included. The exclusion criteria were smoking, pregnancy, patients with autoimmune and infectious diseases, cancer, liver disease, acquired immune deficiency syndrome (AIDS), those taking antithrombotic drugs, prebiotics, probiotics or symbiotic supplements, antioxidants, or the usual intake of cranberry supplements or juice.

Demographic and clinical data, such as the use of medications and physical activity practices, were obtained from patients' medical records and interviews during appointments. Patients received, for over six months, a controlled prescribed diet for CKD, characterized by a low-protein diet (0.6 g/kg/day), low in sodium and adjusted according to the patient's needs, and were then followed up at the outpatient clinic and included in the research. The 24 h dietary recall (24HR) technique was used to assess the estimated energy and macronutrient intake, as described previously [34]. Micronutrient analyses were performed using food composition tables in Brazil [35].

### 2.2. Study Design

This was a double-blind, randomized, placebo-controlled clinical trial in which nondialysis patients with CKD (stages 3-4) were enrolled. After randomization, patients were allocated either to the “placebo group” or “cranberry group” and received two bottles containing 60 capsules each. Patients were instructed to take cranberry capsules twice a day (1 after lunch and 1 after dinner), totaling 1000 mg/day, for two months, or capsules of placebo-500 mg containing corn starch twice daily for the same period. The supplementary material is a flowchart diagram which summarizes the study design with information about included and excluded population and intervention groups.

Adherence to supplementation was verified by weekly telephone contacts and the number of remaining capsules in the delivered bottles. The number of capsules used to calculate adherence was subtracted from the number of capsules dispensed, divided by the number of capsules that patients should have taken for two months, and multiplied by 100 to obtain the adherence percentage. Patients were considered adherent if they consumed >80% of the capsules. The investigators, study participants, and data assessors were blinded to treatment. Biochemical, food intake, and anthropometric data have been detailed previously [34].

After the postintervention results between cranberry and placebo supplementation, we estimated a statistical power greater than 80% (BMI, NF-kB, NRF2, MDA, eGFR, CRP, and IL-6 levels), assuming paired two-tailed *t*-tests with significance levels (type I error probability) of 0.05, and a sample size of 11–23 (in each group) for the calculation of Cohen's d effect sizes. Analyses were performed using R software v.4.2. and package “pwr.”

The local Ethics Committee of Faculdade de Medicina from Universidade Federal Fluminense, Hospital Universitário Antônio Pedro (number CAAE: 85406018.0.0000.5243) approved this study, and all patients provided written informed consent to participate. This trial was registered at ClinicalTrials.gov (NCT04377919).

#### 2.2.1. Primary and Secondary Outcomes

The primary objective was to evaluate the effect of cranberry extract on the mRNA expression of transcription factors involved in inflammation. The secondary outcome was the effect of the cranberry extract on the plasma levels of cytokines and lipid peroxidation markers.

### 2.3. Cranberry Supplement Investigational Product Information

A commercially available cranberry dietary supplement was used according to the label, providing 500 mg of Pacran® per capsule. The PAC content in Pacran® was specified as >1.5% (by high-performance liquid chromatography (HPLC)), with 36 mg of A-type proanthocyanins). For the placebo, the capsules were similar in shape and package, and the composition was gelatin and cornstarch (500 mg of each capsule).

### 2.4. Blood Sample Collection and Biochemical Analysis

Blood samples were collected in the morning, after 12-h fasting, in Vacutainer® tubes containing ethylenediaminetetraacetic acid (EDTA) with anticoagulant (1.0 mg/mL). After collection, an aliquot was used for whole blood analysis to obtain peripheral blood mononuclear cells (PBMC), according to Cardozo et al. [36]. The samples were centrifuged at 2500 rpm for 10 min at 4°C to obtain the plasma, distributed in polypropylene microtubes of 1.5 mL, identified, aliquoted for each analysis, and stored at −80°C for further analysis.

Plasma C-reactive protein (CRP) levels were measured using the immunoturbidimetric method (Quibasa-Bioclin®, Brazil). Other biochemical parameters, such as urea and creatinine, were also measured, as described previously [34]. According to the manufacturer's instructions, plasma inflammatory cytokine IL-6 was measured (duplicate) by commercial ELISA kits (R&D Systems®). The eGFR was calculated using the chronic kidney disease epidemiology collaboration (CKD-EPI) formula [37].

### 2.5. Lipid Peroxidation (Oxidative Stress)

Lipid peroxidation was evaluated using the modified Ohkawa method [38] which measures thiobarbituric acid reactive substances (TBARS) and malondialdehyde (MDA) reactions. Plasma samples (triplicate) were diluted with thiobarbituric acid (0.6% w/v), SDS (8.1% w/v), and phosphoric acid (1% w/v) and then heated at 95°C for 60 min. The microtubes were centrifuged at 4000 rpm for 20 min at 20°C, the supernatant was separated, and the absorbance was measured on a Synergy H1M (BioTek) microplate reader at 532 nm. Plasma levels of TBARS are expressed as nanomoles per mL.

### 2.6. Quantitative Real-Time Polymerase Chain Reaction (qRT-PCR) Analysis

The mRNA expression of NF-kB and Nrf2 was evaluated in PBMC using quantitative real-time polymerase chain reaction (qRT-PCR) according to Cardozo et al. [36]. TaqMan Gene Expression (Thermo Fisher®) assays were used for the detection of Nrf2 mRNA (Hs00975961_g1) and NF-kB (Hs00765730_m1) and the GADPH control gene. The Prism 7500 Sequence Detection System (Applied Biosystems) and standard cycling conditions were used for PCR amplification. NRf2 and NF-kB mRNA expression were normalized against GAPDH, and the expression level was calculated using the ΔΔCt (delta threshold cycle) method.

### 2.7. Statistical Analyses

The Kolmogorov–Smirnov test was used to analyze the distribution of variables. Continuous numerical data are expressed as mean ± SD (standard deviation) or median and interquartile interval, and categorical/nominal data are expressed as absolute and relative proportions. Pearson's correlation coefficient was used to compare continuous variables. The mRNA expression of Nrf2 and NF-kB and cytokine plasma levels were used as outcome variables and log-transformed (base 2 for the mRNA expressions and base 10 for serum levels) as indicated. We also note that any transformation applied to the data is reported in the labels of the graphs to which these log-transformed variables are applied, including their bases. Multiple linear mixed-effects models were used to evaluate time-intervention interactions and patients were considered to have a random effect. Similarly, multiple linear fixed-effects models evaluated the differences between the end (two months) and baseline times. As with the multiple linear mixed-effects models, the fixed systematic component of the models was adjusted for confounding variables (i.e., sex, age, systemic arterial hypertension (SAH), and diabetes mellitus (DM]). The results are presented graphically for the estimated mean marginal effects and 95% confidence intervals. All other variables in the multiple linear mixed models were kept at their mean values or equal proportions and, by contrast, constructed from these estimated mean marginal effects. Tukey's honestly significant difference (HSD) method was used to correct *p* values based on the number of comparisons. Statistical significance was set at *p* < 0.05. The software R version 4.1.1, packages “lME4,” “emmeans,” and their dependencies were used to perform the statistical analyses.

## 3. Results

### 3.1. Characteristics of the Patients

Although 446 patients were assessed for eligibility, most did not meet the inclusion criteria mainly because they were older. Therefore, 30 patients were randomly selected for the cranberry or placebo groups, and 25 participants completed the intervention, as described in detail in our previous study [34].

The placebo group comprised 13 patients (58.9 ± 5.1 years and BMI of 29.8 ± 5.4 kg/m^2^), while the cranberry group comprised 12 patients (56.7 ± 7.5 years and BMI of 29.6 ± 5.5 kg/m^2^). In addition, 24 and 9 patients had hypertension and type 2 diabetes, respectively. Treatment adherence was observed in 96.8% and 98.2% of the patients in the cranberry and placebo groups, respectively. No adverse events were reported in any of the groups. None of the patients reported practicing any physical activity regularly, which did not change during the intervention.

Regarding medication, 96% of the patients used antihypertensive drugs. The following classes of antihypertensive drugs were used by the participants in descending order: angiotensin II receptor blockers (77%), followed by diuretics (61%), beta-blockers (38%), calcium channel blockers (38%), alpha-2 adrenergic receptor agonists (27%), vasodilators (15%), and angiotensin-converting enzyme inhibitors (11%). Lipid-lowering agents, xanthine oxidase inhibitors, hypoglycemic agents, and sodium bicarbonate were prescribed to 74%, 40.6%, 33.3%, and 25.8% of the patients, respectively. The patients did not change their prescriptions during the study period.  Table 1 presents the results of food intake analysis.

### 3.2. Transcription Factors and Inflammatory Parameters

**Figure 1:**
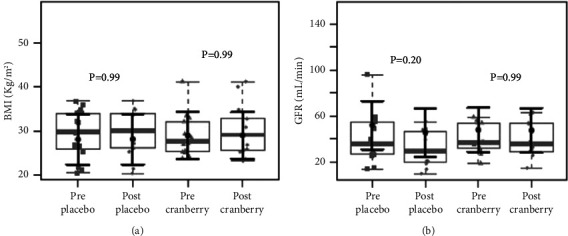
Body index mass (BMI) (a) and glomerular filtration rate (GFR) (b) before and after two months of placebo and cranberry supplementation. There were no differences in body index mass (BMI) (a) and glomerular filtration rate (GFR) (b) after two months of intervention in both groups. The sample distributions of data in grey are represented in boxplots and strip plots. In black, the central circle represents the mean expected marginal effect for each group estimated from linear mixed-effects models. The fixed effects in the models were the intervention group, the time, its interaction, and the confounding variables: sex, age, systemic arterial hypertension (SAH), and diabetes mellitus (DM). Individuals were included as a random effect. Black horizontal bars represent the 95% confidence intervals of the expected mean marginal effects by the group. *p* values were corrected for the number of contrasts/two-by-two comparisons by Tukey's honest significant difference (HSD) method.

**Figure 2:**
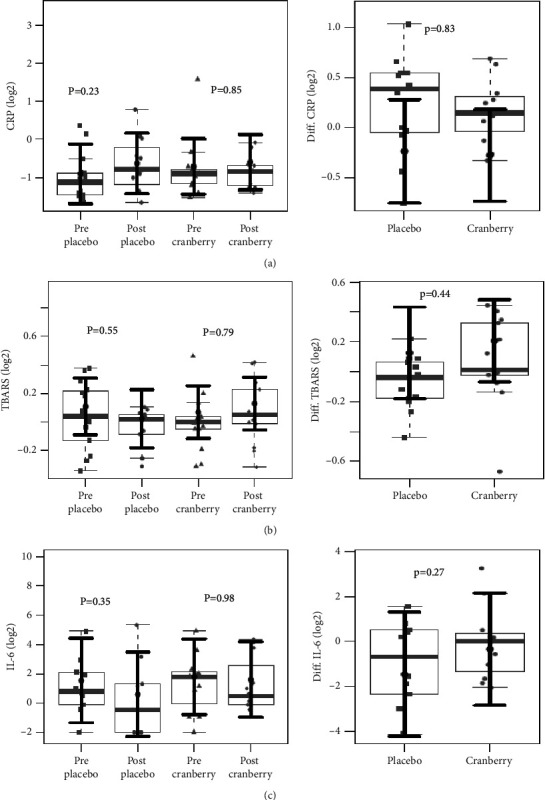
C-reactive protein (CRP) (a), thiobarbituric acid reactive substances (TBARS) (b), and interleukin-6 (IL-6) (c) plasma levels and their respective differences before and after two months of placebo and cranberry supplementation. We found no evidence of C-reactive protein (CRP) (a), thiobarbituric acid reactive substances (TBARS) (b), and interleukin-6 (IL-6) (c) plasma level differences after intervention in cranberry and placebo groups. The sample distributions of data in grey are represented in boxplots and strip plots. In black, the center circle represents the mean expected marginal effect for each group estimated from linear mixed-effects models. The fixed effects in the models were the intervention group, the time, its interaction, and the confounding variables sex, age, systemic arterial hypertension (SAH), and diabetes mellitus (DM). Individuals were included as a random effect. Black horizontal bars represent the 95% confidence intervals of the expected mean marginal effects by the group. *p* values were corrected for the number of contrasts/two-by-two comparisons by Tukey's honest significant difference (HSD) method.

**Figure 3:**
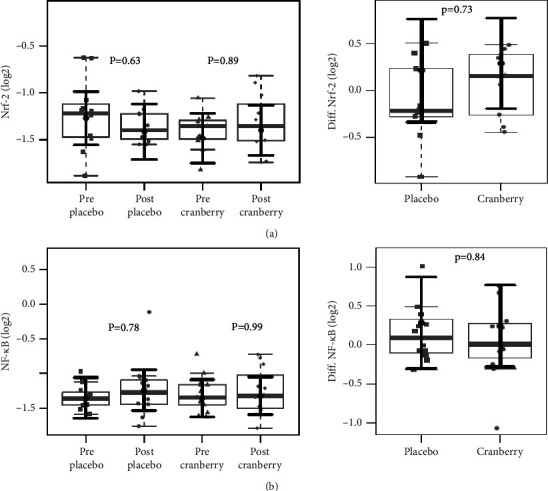
mRNA expression of nuclear factor erythroid 2-related factor-2 (Nrf2) (a) and nuclear factor-kappa B (NF-*κ*B) and their respective differences before and after two months of placebo and cranberry supplementation. There was no evidence of mRNA expression of nuclear factor erythroid 2-related factor-2 (Nrf2) (a) and nuclear factor-kappa B (NF-*κ*B) (b) differences after intervention in cranberry and placebo groups. The sample distributions of data in grey are represented in boxplots and strip plots. In black, the center circle represents the mean expected marginal effect for each group estimated from linear mixed-effects models. The fixed effects in the models were the intervention group, the time, its interaction, and the confounding variables: sex, age, systemic arterial hypertension (SAH), and diabetes mellitus (DM). Individuals were included as a random effect. Black horizontal bars represent the 95% confidence intervals of the expected to mean marginal effects by the group. *p* values were corrected for the number of contrasts/two-by-two comparisons by Tukey's honest significant difference (HSD) method.

There were no significant differences in the baseline anthropometric and biochemical variables between the groups [34].  Figure 1 shows that the BMI and GFR values did not change after the intervention. The plasma levels of CRP, TBARS, and IL-6 and their respective differences did not change after the intervention, as shown in Figure 2. In addition, neither group differed in the mRNA expression of Nrf2 and NF-kB (Figure 3).

## 4. Discussion

The present study investigated, for the first time, whether cranberry (Pacran) supplementation could modulate the expression of the transcription factors NF-kB and Nrf2, the inflammatory markers (IL-6 and CRP), and lipid peroxidation (TBARS) in patients with CKD. However, our results showed that cranberry supplementation did not alter the mRNA expression of NF-kB or Nrf2, lipid peroxidation, or inflammation in patients with CKD (stages 3 and 4).

Several studies have used bioactive compounds obtained from food as a complementary therapy in CKD treatment because of their many benefits, including antihypertensive properties, amelioration of lipoprotein profile, and antioxidant and anti-inflammatory effects through the modulation of transcription factors involved in inflammation and oxidative stress, which can slow CKD progression [15, 17, 18, 23, 27]. Advanced stages of CKD usually present with altered transcription factor expression, indicating higher NF-kB mRNA expression and decreased Nrf2 mRNA expression compared with healthy individuals [39]. Therefore, the role of bioactive compounds in CKD in modulating these factors and reducing the inflammatory response is being studied [26, 36, 40]. We previously showed that Brazilian nut supplementation in patients with CKD undergoing hemodialysis reduced NF-kB expression and increased Nrf2 activation [36]. Also, oral curcumin supplementation reduced NF-kB expression and CRP levels in hemodialysis patients [26].

Although studies on patients with CKD are scarce, experimental studies have reported beneficial effects. Caco-2/15 intestinal cells treated with cranberry polyphenols showed a reduction in NF-kB activation, stimulation of Nrf2 activation, and increased antioxidant enzyme production [33]. Likewise, diet-induced metabolic syndrome mice treated with cranberry extract normalized the NF-*κ*B/I*κ*B ratio [30]. However, our results did not reveal any modulation of these transcription factors. Additional preclinical and clinical studies are required to evaluate the effects of cranberry polyphenol metabolites and their roles in inflammation.

Cranberry polyphenols and their derived metabolites are considered prebiotic compounds because they reach the colon and are metabolized into organic acids by the gut microbiota, thus becoming available for absorption and exerting health benefits [41]. They can alter the components of the gut microbiota, affecting the function and production of secondary metabolites from dietary ingestion [42]. Furthermore, polyphenols interact with cell membranes through covalent, electrostatic, or hydrophobic bonds, altering their organization and function, which can benefit the host [43]. Some studies have described the beneficial effects of polyphenols through the regulation of cell signaling because they can regulate gene expression transcriptionally and posttranscriptionally via microRNAs [44].

The potential benefits of cranberries in relieving hepatic inflammation, dyslipidemia, oxidative damage, and liver steatosis are achieved through diverse mechanisms, such as the reduction of lipid peroxidation and inflammatory cytokines, modulation of transcription factors, and reversion of the expression of proinflammatory microRNAs [45–47]. Despite varied doses of cranberries and different models, most studies have found positive results, whereas clinical studies are controversial.

In contrast to our study population, most clinical trials with cranberries included healthy individuals and fewer evaluated volunteers with diabetes or metabolic syndrome, and cranberries were reported to have beneficial or neutral effects on oxidative stress, dyslipidemia, and inflammatory markers [48–53].

Supplementation of cranberry juice (480 mL/day, for eight weeks) in women with metabolic syndrome reduced oxidized low-density lipoprotein and the lipid peroxidation marker, malondialdehyde (MDA). However, there were no changes in CRP and IL-6 levels [53]. Similarly, cranberry juice (700 mL/day for 60 days) in patients with metabolic syndrome reduced lipid peroxidation and protein oxidation levels but did not reduce TNF-*α*, IL-1, and IL-6 [54]. Patients with diabetes who received 240 mL of cranberry juice for 12 weeks showed lower serum glucose levels and improved levels of cardiovascular risk markers (apoB, apoA-1, and Paraoxonase-1) [55]. Another study on healthy subjects who ingested cranberry juice showed reduced plasma CRP levels, while no proinflammatory cytokines were altered [51].

The present study included patients with CKD, which also presents with other associated comorbidities, such as diabetes and hypertension; however, our findings did not show any reduction in lipid peroxidation, CRP, or IL-6. Furthermore, our previous study did not find improvements in the lipid profile or modulation of lipopolysaccharide (LPS) or plasma uremic toxin levels in these patients [34]. The complexity of CKD may require a higher dose of cranberry extract or a different supplementation, which should be tested in future research. Furthermore, most studies used cranberry juice, although the most common cranberries used by the population are extracts and powder nutraceutical supplements [56]. Therefore, this study is relevant because it evaluated a commonly prescribed commercial cranberry supplement.

Interpreting polyphenol findings in cranberry studies is complex and requires consideration of various cranberry-derived products. Moreover, several individual factors may influence the metabolism, absorption, interaction, and effects of polyphenols, such as genetic polymorphisms of enzymes and transporters, age, sex, the presence of diseases, diet, modulation of transcription factors, variation in gut microbiota, and interactions between the microbiota and miRNAs [57]. Only a few studies have investigated plasma polyphenol dose-response profiles after the intake of polyphenol-rich supplements or food, and there is only a linear dose-response among the distinctive polyphenols. A study evaluating cranberry polyphenol metabolism in humans reported substantial interindividual variations in metabolic profiles, probably due to variations in the gut microbiome [58]. Moreover, another study reported low plasma cranberry polyphenol bioavailability and interindividual differences in patients with coronary artery disease. It has been suggested that it could be insufficient to promote free radical neutralization directly depending on the polyphenol concentration. However, cranberries can indirectly increase antioxidant enzyme production by modulating gene expression [59]. However, the cell signaling pathways affected by polyphenols remain poorly understood, and additional scientific evidence is required to support these mechanisms.

To the best of our knowledge, the potential effects of cranberry supplementation on inflammation and oxidative stress in patients with CKD are yet to be investigated in prospective longitudinal studies. In addition to the present study, further studies should be performed because of the beneficial cranberry properties described in the literature.

The interpretation of the results presented here has some limitations. The varied data on cranberry dose, type, and time of supplementation make it difficult to affirm whether the dose and time of supplementation used were sufficient to promote the benefits of cranberries in patients with CKD, a very complex disease. We evaluated cranberry extract supplementation, as it is one of the most widely used and easiest to access in our population. However, only some studies have examined the effect of this type of supplementation on UTI. Reviews have shown that cranberry supplements have varying PAC concentrations, which are rarely measured or standardized. Another limitation was the power calculation, which was performed as a post hoc analysis because there were no studies in patients with CKD.

This well-conducted study had several strengths. No adverse effects were observed with supplementation, and the participant dropout rate was low; however, cranberry extract supplementation had no positive results on the parameters evaluated here. The present study, along with [34], is the first clinical trial conducted using cranberries in patients with CKD. However, most studies that included cranberry supplements excluded these patients. Therefore, considering the typical application of cranberry supplementation for UTI prevention, which is frequently observed in this population, we hypothesized that cranberries could be beneficial in reducing oxidative stress and inflammation.

## 5. Conclusion

In conclusion, supplementation with cranberry (Pacran) for two months may not mitigate inflammation or modulate the expression of the transcription factors NF-kB and Nrf2 in patients with CKD. Although Pacran supplementation in patients with CKD did not affect the evaluated inflammatory and oxidative stress markers, further investigations are necessary with higher amounts of PACs.

## Figures and Tables

**Table 1:** Food intake analysis obtained from the 24 h dietary recall of placebo and cranberry groups presupplementation and postsupplementation.

Energy and nutrients	Pre	Post	*p* value	Pre	Post	*p* value
	Placebo			Cranberry		
Energy intake (kcal/d)	912.3 ± 309.3	853.1 ± 309.3	0.96	1024.9 ± 272.8	1103.1 ± 272.8	0.87
Protein (g/d)	43.3 ± 15.6	39.2 ± 15.6	0.98	37.2 ± 13.8	44.8 ± 13.8	0.86
Lipids (g/d)	18.8 ± 11.7	16.1 ± 11.7	0.94	20.7 ± 10.3	25.7 ± 10.3	0.66
Carbohydrate (g/d)	142.5 ± 50.6	137.8 ± 50.6	0.99	172.5 ± 44.6	173.3 ± 44.6	0.99
Fibre (g/d)	12.4 ± 7.1	12.9 ± 7.1	0.99	20.8 ± 6.3	15.5 ± 6.3	0.44
Cholesterol (mg/d)	133.6 ± 75.8	153.9 ± 76.2	0.98	127.4 ± 68.6	207.3 ± 63.9	0.22
Calcium (mg/d)	112.7 ± 117.7	121.9 ± 117.6	0.99	266.4 ± 102.8	269.7 ± 102.8	0.99
Magnesium (mg/d)	103.9 ± 40	99.8 ± 40	0.99	140.6 ± 35.2	132.3 ± 35.2	0.96
Manganese (mg/d)	1.9 ± 0.6	1.3 ± 0.6	0.56	2.1 ± 0.5	1.6 ± 0.5	0.48
Phosphorus (mg/d)	477.2 ± 186.1	430.3 ± 186.1	0.95	469.5 ± 164.1	530.9 ± 164.1	0.85
Iron (mg/d)	3.2 ± 1.3	3.7 ± 1.3	0.91	4.1 ± 1.1	3.9 ± 1.2	0.98
Sodium (mg/d)	543.1 ± 527.1	499.3 ± 535.6	0.99	918.3 ± 465.8	914.3 ± 465.7	1.00
Potassium (mg/d)	981.1 ± 393.8	970.9 ± 393.8	1.00	1406.1 ± 347.1	1523.7 ± 347.1	0.91
Copper (mg/d)	0.4 ± 0.2	0.4 ± 0.2	0.99	0.5 ± 0.1	0.5 ± 0.1	0.99
Zinc (mg/d)	3.0 ± 1.7	4.1 ± 1.7	0.65	4.0 ± 1.5	4.9 ± 1.5	0.99
Retinol (mg/d)	69.2 ± 46.9	76.3 ± 49.4	0.99	65.3 ± 44.5	103.8 ± 41.3	0.45
Thiamine (mg/d)	1.9 ± 1.1	0.4 ± 1.1	0.45	1.1 ± 1.0	0.7 ± 1.0	0.96
Riboflavin (mg/d)	0.6 ± 0.2	0.3 ± 0.2	0.45	0.6 ± 0.2	0.3 ± 0.2	0.32
Pyridoxine (mg/d)	0.7 ± 0.2	0.5 ± 0.2	0.75	0.6 ± 0.1	0.5 ± 0.1	1.00
Niacin (mg/d)	7.2 ± 2.8	6.6 ± 0.2.7	0.99	6.2 ± 2.4	7.3 ± 2.4	0.93
Vitamin C (mg/d)	44.6 ± 66.6	37.3 ± 65.0	0.99	79.0 ± 63.0	79.5 ± 55.3	1.00

Data are presented as mean ± standard error.

## Data Availability

The data that support the findings of this study are available in Google Drive at https://docs.google.com/spreadsheets/d/1rD8okISyQUlxkZRSEHNkEOhxiJZ2jMe7/edit?usp%3Dsharing%26%3Bouid%3D100967213521687822596%26%3Brtpof%3Dtrue%26%3Bsd%3Dtrue.

## Supplementary Materials

Supplementary Materials Flowchart summarizing the study design with information about included and excluded population and intervention groups.
