# Supplementary material for: Effects of Cranberry Extract (Vaccinium macrocarpon) Supplementation on Lipid Peroxidation and Inflammation in Patients with Chronic Kidney Disease (Stages 3-4): A Randomized Controlled Trial
Source: J Nutr Metab. 2024 May 8;2024:9590066. doi: 10.1155/2024/9590066 (PMC11095989; doi:10.1155/2024/9590066)
Supplement: Supplementary Materials — Flowchart summarizing the study design with information about included and excluded population and intervention groups. [file 9590066.f1.docx]

4 men / 8 women

Intervention

Age: 56.7 ± 7.5 years,

Body mass index (BMI):

29.6 ±5.5 kg/m²

1000 mg of placebo

(corn starch)/day

1000 mg of cranberry

extract/day

Placebo

Results

1 capsule after lunch and 1 after dinner

for 2 months

Inclusion and Exclusion Criteria

Randomisation

Study

Sample

Study

population

**Inclusion**

- Non-dialysis (stages 3-4) chronic kidney disease (CKD) patients
- 20-65 years;

4 men / 9 women

Age: 58.8 ± 5.1 years, BMI: 29.8±5.4 kg/m²

**Exclusion**

- Pregnancy;
- Antibiotics, antioxidant, cranberry supplements,
- Prebiotic, probiotic, symbiotic supplements.
- Autoimmune and infectious diseases;
- Cancer, liver diseases, and acquired human immunodeficiency syndrome (AIDS)
